# Bone Regeneration of Rat Tibial Defect by Zinc-Tricalcium Phosphate (Zn-TCP) Synthesized from Porous *Foraminifera* Carbonate Macrospheres

**DOI:** 10.3390/md11125148

**Published:** 2013-12-16

**Authors:** Joshua Chou, Jia Hao, Shinji Kuroda, David Bishop, Besim Ben-Nissan, Bruce Milthorpe, Makoto Otsuka

**Affiliations:** 1 Research Institute of Pharmaceutical Sciences, Faculty of Pharmacy, Musashino University, 1-1-20 Shin-machi, Nishitokyo-Shi, Tokyo 202-8585, Japan; E-Mail: motsuka@musashino-u.ac.jp; 2 Faculty of Science, University of Technology Sydney, P.O. Box 123, Broadway, Ultimo, Sydney, NSW 2007, Australia; E-Mails: David.Bishop@uts.edu.au (D.B.); Besim.Ben-Nissan@uts.edu.au (B.B.-N.); Bruce.Milthorpe@uts.edu.au (B.M.); 3 Oral Implantology and Regenerative Dental Medicine, Tokyo Medical and Dental University, 1-5-45 Yushima, Bunkyo-ku, Tokyo 113-8510, Japan; E-Mails: haoirm@tmd.ac.jp (J.H.); skuroda.mfc@tmd.ac.jp (S.K.)

**Keywords:** biomimetic, zinc-tricalcium phosphate, foraminifera, bone defect, bone regeneration

## Abstract

*Foraminifera* carbonate exoskeleton was hydrothermally converted to biocompatible and biodegradable zinc-tricalcium phosphate (Zn-TCP) as an alternative biomimetic material for bone fracture repair. Zn-TCP samples implanted in a rat tibial defect model for eight weeks were compared with unfilled defect and beta-tricalcium phosphate showing accelerated bone regeneration compared with the control groups, with statistically significant bone mineral density and bone mineral content growth. CT images of the defect showed restoration of cancellous bone in Zn-TCP and only minimal growth in control group. Histological slices reveal bone in-growth within the pores and porous chamber of the material detailing good bone-material integration with the presence of blood vessels. These results exhibit the future potential of biomimetic Zn-TCP as bone grafts for bone fracture repair.

## 1. Introduction

Throughout time, bone fracture has continued to impact societies, increasing socioeconomic burden to patients. While advancement in medical technologies has helped in the recovery process, it is expected by society that improvements in treatment are continually made. This has sparked an era of development in innovative biomaterial and opened the field of bone tissue engineering, in order to address this ongoing trauma. In simple terms, the goal is to develop more biologically active materials that can reduce the recovery time thereby reducing the costs and patient discomfort. Currently, there is a wide range of materials that are available clinically, and amongst these, calcium phosphate bioceramics have been extensively studied and developed as bone grafts. While these byproducts can “do the job”, the bone repair process is a complex system with many factors contributing to its growth. This has led biomaterial scientists to take on a biomimetic approach, looking towards nature for alternative materials. With all the discoveries in the vast ocean, the marine world has barely been uncovered, and many prospective and potentially beneficial materials have yet to be discovered. In our previous studies, *foraminifera* calcareous exoskeleton was identified to possess uniformly and interconnected pores capable of stimulating key bone remodeling cells [1] once hydrothermally converted to calcium phosphate. Furthermore, the material was shown to also be an effective drug delivery carrier for bone stimulating drugs [2,3,4,5]. Hydrothermal conversion, first developed in 1974 [6], allows the carbonate within the material to be substituted with phosphate ions thereby making it more biocompatible and biodegradable [7,8]. It should be noted that the integrity of the original structure is preserved during this process. In this study, we investigated first, the ability to synthetically modify the chemical composition of the material to include zinc ions to produce zinc tricalcium phosphate (Zn-TCP) and subsequently evaluating the bone repair properties of Zn-TCP in a rat tibial defect model. Key bioinorganic ions such as strontium and magnesium are commonly incorporated into biomaterials to be released over time to supplement and stimulate the bone regeneration process by acting on or in combination with osteoblast and osteoclast cells. Zinc ions, commonly associated with cell regulation, have been shown to affect the processes of physiological bone homeostasis and pathological bone turnover [9]. In humans, zinc intake has been positively correlated with bone mineral content in premenopausal women [10], whereas zinc deficiency has been linked to growth retardation and hypogonadism [11], conditions that affect skeletal maturation and integrity. Zinc is a nutritional factor that has long been recognized as important for prenatal and postnatal skeletal development, whereas supplementation ameliorates pathological bone loss [9]. Zinc differentially regulates bone formation and bone resorption promoting an osteoblastic gene program while repressing osteoclast differentiation [12,13,14] leading to a stimulatory effect on bone formation and mineralization *in vitro* and *in vivo* [15,16,17]. These factors have motivated the development of various zinc-tricalcium phosphate materials, which have shown to be effective in treating bone-related ailments and traumas [18,19,20,21]. However, these materials currently lack the interconnected porous network features of natural biomaterials and the synthesis of Zn-TCP requires high temperature sintering around 800–1000 °C [18]. With these in mind, this study is aimed to develop an alternative Zn-TCP material capable of improving the repairs of bone defects. 

## 2. Results and Discussion

### 2.1. Physico-Chemical Characterization of Zn-TCP

One of our key aims is to synthesize Zn-TCP using *foraminifera* macrospheres as a precursor material and evaluating the biological and bone healing response in a rat tibial defect. The production of Zn-TCP is based on hydrothermal conversion in which the carbonate component is replaced with phosphate ions while preserving the architectural structure of the material that can be potentially beneficial in bone regeneration. Our data shows Zn-TCP can be produced by hydrothermal conversion where zinc is substituted for calcium ions and this can be achieved at relatively low temperature (220 °C) compared with sintering.

#### 2.1.1. X-Ray Powder Diffraction (XRD) Analysis

**Figure 1:**
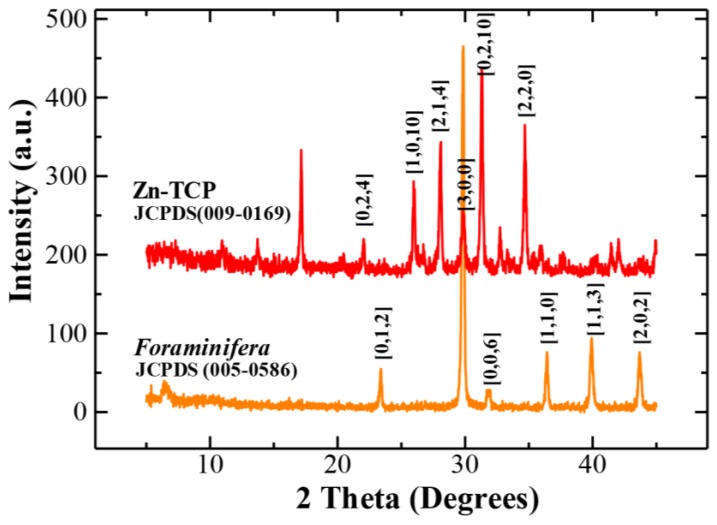
XRD peak patterns matched *foraminifera* calcareous (aragonite) material and hydrothermally synthesized zinc tricalcium phosphate (Zn-TCP) from JCPDS database.

The replacement of carbonate ions with phosphate was verified crystallographically by XRD (Figure 1), which showed peak patterns matching tricalcium phosphate. It can be seen that the peaks of Zn-TCP does not differ much from that of tricalcium phosphate as the amount of zinc incorporated is at relatively low concentration (~5%). 

#### 2.1.2. Quantitative Measurement of Ionic Composition of Samples

Just like with the use of any pharmaceutical compounds, the therapeutic efficacy of zinc-related materials is highly dependent on the concentration. Supporting studies have shown that the most ideal and optimal concentration is around the 5% range where excess amounts can induce inflammatory side effects [21]. The key chemical compositions of the materials (*foraminifera*, β-TCP and Zn-TCP) was quantified by mass spectroscopy and presented in Table 1. From the table, it can be seen that phosphate ions were successfully introduced into the material following hydrothermal conversion and calcium concentration was retained at similar levels. Quantitative measurements by mass spectroscopy showed the zinc concentration of the synthesized Zn-TCP is approximately 6%, which is within the therapeutic range. 

**Table 1:** Ionic composition of samples.

Materials	Calcium (mg/g)	Phosphate (mg/g)	Zinc (mg/g)
***Foraminifera***	0.043 ± 0.0001	0	0
**β-TCP**	0.045 ± 0.0002	0.0041 ± 0.0001	0
**ZnTCP**	0.05 ± 0.0001	0.0046 ± 0.0001	0.0003 ± 0.00001

#### 2.1.3. Morphological Observation by Scanning Electron Microscopy (SEM)

**Figure 2:**
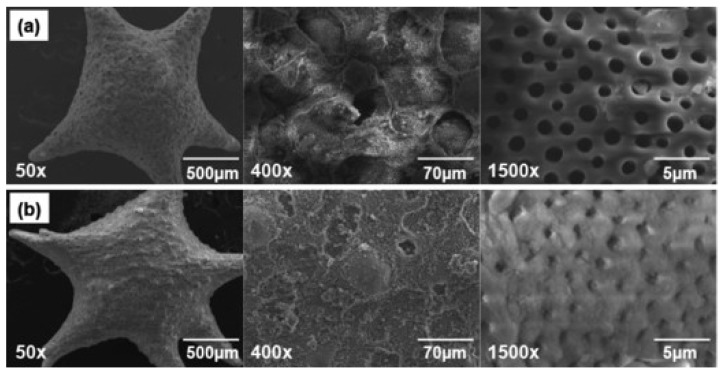
(**a**) *Foraminifera* carbonate material showing unique spherical structure and surface distribution of uniform pores; (**b**) Zn-TCP material showing preservation of the original structure and deposits of zinc ions on the surface structure.

The rationale for using *foraminifera* precursor materials is due to the unique uniform and interconnected pores that the material naturally developed. By using hydrothermal conversion, these traits can be preserved during the transformation process. SEM micrographs presented in Figure 2 reveals a naturally spherical/star shaped material, which upon closer examination, shows the uniformity of the pore (~2 μm in diameter) distribution throughout the surface of the sample. 

This could essentially allow for increase cell infiltration to regenerate bone and the development of crucial blood vessels allowing improved blood flow, supplying the area with essential nutrients. Previous studies by micro-computed tomography have shown that these materials are internally interconnected through the surface pores to a central hub [4]. Moreover, these pores are preserved during the synthesis process and from Figure 2b additional deposits of zinc as determined by energy dispersive spectroscopy (EDS) can be seen scattered across the surface. This can potentially add to the therapeutic value of the material. 

### 2.2. Radiological and Histological Assessment of Bone Regeneration

**Figure 3:**
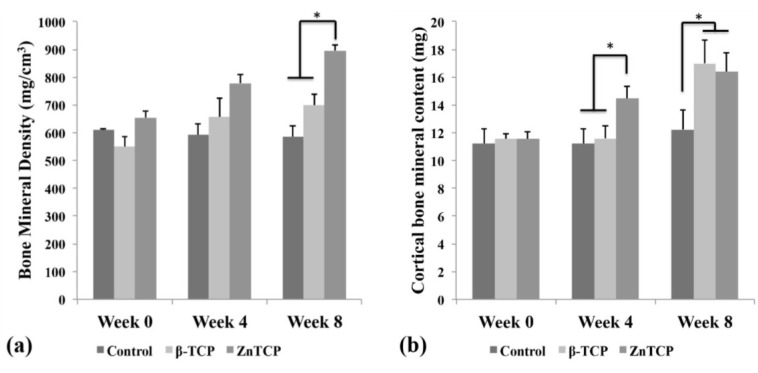
(**a**) Bone material density (BMD) values showing control and β-TCP group maintaining similar levels while Zn-TCP group showed statistical increase after eight weeks; (**b**) bone material content (BMC) which reflect on the new bone formed showed no significant growth in control group while β-TCP saw statistical growth after eight weeks. It was observed that Zn-TCP group showed a faster statistical bone growth after four weeks compared with control and β-TCP. Asterisk sign in Figure 3(**a**) and (**b**) represent *p* < 0.05, which was considered statistically significant.

Zn-TCPs were implanted in a rat tibial defect model for eight weeks to evaluate the material’s bone healing properties. Zn-TCP samples were compared with empty control and β-TCP as a benchmark to determine the effectiveness of zinc in the bone healing process. The bone mineral density (BMD) (Figure 3a) and the bone mineral content (BMC) (Figure 3b) were calculated based on X-ray measurements and summarized in Figure 3. It should be noted that the BMD and BMC values took into account the presence of the sample material and that these were included in the results. The BMC values includes both cortical and trabecular bone amount. 

The BMD of the samples did not show any significant differences until eight weeks where Zn-TCP reached a statistically higher level compared with empty control and β-TCP. The complementing BMC data showed that Zn-TCP achieved a statistically higher level of bone mineral formation at four weeks compared with the control groups. This trend was also observed at eight weeks but with both β-TCP and Zn-TCP showing similar bone mineral levels compared with the significantly lower BMC of the empty control group. This result is in agreement with our hypothesis that β-TCP would have some degree of stimulatory action on bone formation as calcium and phosphate ions are released as the material degrades, thereby supplementing the defect area. Zn-TCP group was able to achieve a statistically higher bone mineral level as early as four weeks and was the only experimental group to show a significant increase in BMD levels. This indicates that Zn-TCP was able to stimulate faster bone mineral formation through the actions of the released zinc ions. While *in vitro* studies have shown zinc to act on osteoblasts and osteoclasts, the exact mechanism of these effects is still poorly understood. It was shown that zinc could increase the osteogenic effect by increasing the osteoblast cell proliferation and stimulating alkaline phosphatase activity and collagen synthesis during the proliferation and differentiation phase [22].

#### 2.2.1. Computed Tomography (CT) Imaging of Bone Regeneration

**Figure 4:**
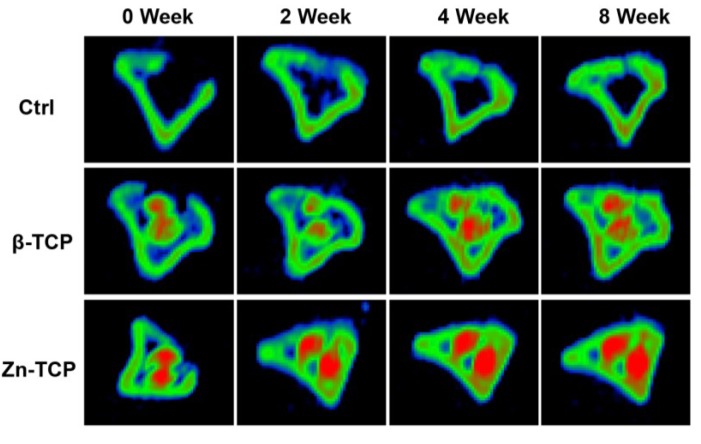
CT images corresponding to the empty control (top), β-TCP (middle) and Zn-TCP (bottom) showing the progressive restoration of cortical and trabecular bone over eight weeks at the defect. Zn-TCP showed complete cortical bone restoration at four weeks and continuous growth of trabecular bone. The sample materials are highlighted in red.

The restoration of bone at the defect was observed using X-ray CT scans detailing the progressive bone regeneration illustrated in Figure 4. The control group showed that the majority of the cortical bone was restored at eight weeks but minimal trabecular bone can be seen within the marrow cavity. The β-TCP group showed at two weeks a thin layer of cortical bone developing at the defect and the progressive restoration of trabecular bone up to eight weeks. The image showed Zn-TCP to completely restore the cortical bone at the defect at four weeks and thickening of the bone can be seen at eight weeks. In addition, it can be seen that the restoration of the trabecular bone in the Zn-TCP group was more mature and dense compared with β-TCP. These results are in agreement with the BMD and BMC data results showed previously. 

#### 2.2.2. Histological Assessment of Defect Site

**Figure 5:**
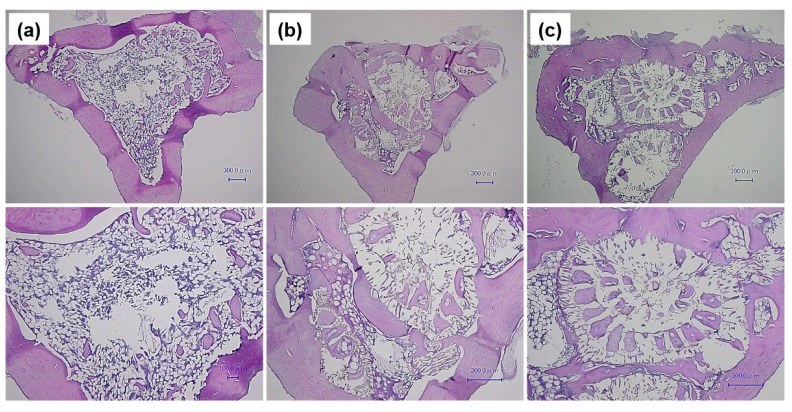
HE-stained bone: (**a**) Control group with bone site regenerated, but, at higher magnification showed minimal cancellous bone growth; (**b**) β-TCP reveals the internal structure of the material with porous chambers penetrated by new formed bone and presence of blood vessels; (**c**) Zn-TCP sample shows similar results to β-TCP, with new bone formed within the porous chamber of the material showing good bone-material integration.

Figure 5 show histological HE-stained slices of (a) control, (b) β-TCP and (c) Zn-TCP with corresponding images shown at higher magnification. From the images it can be seen that both β-TCP and Zn-TCP were able to stimulate regeneration of new bone closing the defect, but were also regenerating cancellous bone. The empty control group only showed a very small amount of regenerated cancellous bone. The higher magnification images reveal the internal porous chambers of the β-TCP and Zn-TCP material showing new bone in-growth penetrating these porous networks. The ability of the biomaterial to promote bone infiltration is crucial in generating good integration between the material and its surrounding environment. Upon closer observation of these porous chambers, the presence of blood vessels with the newly formed bone could be observed suggesting good blood flow allowing the required nutrients to aid in the regeneration process. 

These results may have significant implication in bone fracture repair and it is hoped this can provide the background for future studies in the development of more bioactive biomaterials for bone fracture repair. Current and future studies will examine the activity of Zn-TCP in larger animal models and in critical-sized defect models. 

## 3. Experimental Section

### 3.1. Production, Synthesis and Evaluation of Zn-TCP

The *foraminifera* carbonate samples were commercially acquired (Okinawa Business Support Okinawa, Okinawa, Japan) and cleaned for 25 min in sodium hypocholorite to remove any contaminants. The carbonate samples were first hydrothermally converted to tricalcium phosphate using aqueous diammonium hydrogen-phosphate (Wako Chemical Co., Tokyo, Japan) with Ca/P molar ratio adjusted to 1.5, then heating the specimen in a high pressure vessel to 220 °C for 48 h. The aqueous solution was replaced with aqueous zinc nitrate hexahydrate solution (Wako Chemical Co., Tokyo, Japan) with Ca/Zn ratio adjusted to 19/2 to produce 5% Zn-TCP samples. The samples underwent another hydrothermal treatment again by heating to 220 °C for 48 h to produce Zn-TCP. The crystallographic characterization was performed by powder X-ray diffraction analysis (RINT-Ultima-III, Rigaku Co., Tokyo, Japan; CuKα radiation, 40 kV, 40 mA) with peak patterns matched with JCPDS database. The surface morphological structures of all the specimens were observed using a scanning electron microscope (JEOL JSM-7600F, Field Emission SEM, 10 kV, Tokyo, Japan). The *in vitro* dissolution kinetic studies were evaluated in a previously published study [1]. The ionic compositions of the samples were quantified by inductively coupled plasma-mass spectroscopy (ICP-MS) using an Agilent Technologies 7500 ce series ICP-MS (Agilent, Sydney, Australia). Sample introduction was made via a micromist concentric nebuliser (Glass expansion) and a Scott type double pass spray chamber cooled to 2 °C. The sample solution and the spray chamber waste were carried with the aid of a peristaltic pump. The ICP operating parameters and the lens conditions were selected to maximise the sensitivity of a mixture of 1% HNO_3_, 1% HCl solution containing 1 ng/mL of Li, Co, Y, Ce and Tl. Helium was added into the octopole reaction cell to reduce interference. Calibration curves were constructed and the results analysed using Agilent Technologies Masshunter software. Approximately 0.005 g of sample was digested with 0.25 mL of 1%HNO_3_. Once the digestion was completed, the sample volume was made up to 5 mL with water. The samples underwent a further 1:100 dilution with a 1% nitric acid solution before ICP-MS analysis. Samples were diluted further in nitric acid as required.

### 3.2. Animal Maintenance and Surgical Procedures

Animal care and studies were in accordance to the guidelines and approval from the Animal Ethics Committee at Musashino University, Japan. Thirty, 15-week-old Wistar male rats were randomly and equally divided into three groups: (1) Control (Ctrl); (2) beta-tricalcium phosphate (β-TCP); and (3) zinc-tricalcium phosphate (Zn-TCP). The animals were monitored on a daily basis for signs of inflammation during the early stages of recovery and their weights were recorded on a weekly basis to ensure normal growth of the animal. The rats were initially anesthetized intramuscularly using a combination of ketamine (40 mg/kg) and xylazine (4 mg/kg). The area around the incision site was shaved and disinfected with alcohol and an incision of approximately 1 cm long was made to expose the tibia where a 2 mm in diameter and 3 mm in depth cylindrical defect was made using a fissure bur under constant saline irrigation. Defects were either left empty (control group) or filled with two β-TCP and ZnTCP samples. The soft tissue was closed in two layers with absorbable sutures and the wound region was disinfected again. The animals were sacrificed after eight weeks and radiologic and histologic analyses were used to assess total bone mineral content and bone mineral density.

### 3.3. Radiological and Histological Assessment of Defect

Radiological assessment of the defect site was made by scanning every two weeks using X-ray computed tomography (X-Ray CT) (Latheta 200, Aloka, Tokyo, Japan) and evaluating changes in bone mineral density and bone mineral content by software (Latheta, Aloka, Tokyo, Japan). At eight weeks, the animals were sacrificed and the tibia was excised and fixed in 10% formalin (Wako Chemicals, Tokyo, Japan). The fixed samples were subsequently decalcified in 5% formic acid (Wako Chemicals, Tokyo, Japan) for four weeks. After decalcification, the samples were dehydrated in ascending grades of ethanol and embedded in paraffin where 5 µm thick coronal sections of the central area at the defect site was cut and prepared for staining with hematoxylin and eosin (H&E). 

### 3.4. Statistical Analysis

Statistical analysis was performed by using SPSS Ver 11.5 for Windows. ANOVA with Scheffe test was used for comparing the significance amongst the groups. *P* values of less than 0.05 were considered to be statistically significant.

## 4. Conclusions

Based on these findings, biomimetic Zn-TCP showed improved restoration of bone in a rat tibial defect model with β-TCP and empty control group. The present study showed that Zn-TCP derived from *foraminifera* carbonate precursor material could stimulate accelerated bone formation compared with β-TCP and empty control when implanted in rat tibial defects. It was also shown that newly formed bone infiltrated the porous chambers of the material and the presence of blood vessels was observed within these chambers. 
